# *catena*-Poly[[[4,6-bis­(2-pyrid­yl)-1,3,5-triazin-2-olato]copper(II)]-μ-chlorido]

**DOI:** 10.1107/S1600536811016989

**Published:** 2011-05-11

**Authors:** Man-Li Cao

**Affiliations:** a Department of Chemistry, Guangdong University of Education, Guangzhou 510303, People’s Republic of China

## Abstract

The title compound, [Cu(C_13_H_8_N_5_O)Cl]_*n*_, has a chain structure parallel to [100] with Cu^2+^ cations in a trigonal–bipyramidal coordination environment. The ligand adopts a tridentate tripyridyl coordination mode and a chloride ion acts as a bridge. The chains are linked *via* weak C—H⋯O and C—H⋯Cl hydrogen bonds into a three-dimensional supra­molecular network.

## Related literature

For background to rigid multidentate polypyridyl ligands containing a triazine ring as a bridge, see: Zhou, Li, Zheng *et al.* (2006 ▶); Zhou, Li, Wu *et al.* (2006 ▶). For the synthesis of the ligand, see: Wieprecht *et al.* (2005 ▶). For complexes based on the ligand, see: Cao *et al.* (2008 ▶, 2009 ▶).
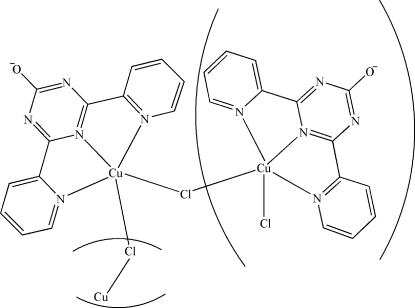


## Experimental

### 

#### Crystal data

[Cu(C_13_H_8_N_5_O)Cl]
*M* *_r_* = 349.23
Monoclinic, 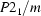

*a* = 3.7687 (13) Å
*b* = 13.698 (5) Å
*c* = 11.852 (4) Å
β = 92.851 (6)°
*V* = 611.1 (4) Å^3^
*Z* = 2
Mo *K*α radiation
μ = 2.01 mm^−1^
*T* = 293 K
0.09 × 0.09 × 0.07 mm

#### Data collection

Bruker SMART APEX CCD area-detector diffractometer
Absorption correction: multi-scan (*SADABS*; Sheldrick, 1996 ▶) *T*_min_ = 0.840, *T*_max_ = 0.872
3063 measured reflections
1109 independent reflections
952 reflections with *I* > 2σ(*I*)
*R*_int_ = 0.030

#### Refinement

*R*[*F* ^2^ > 2σ(*F* ^2^)] = 0.033
*wR*(*F* ^2^) = 0.085
*S* = 1.03
1109 reflections
103 parameters
H-atom parameters constrained
Δρ_max_ = 0.49 e Å^−3^
Δρ_min_ = −0.27 e Å^−3^

### 

Data collection: *SMART* (Bruker, 2005 ▶); cell refinement: *SAINT* (Bruker, 2005 ▶); data reduction: *SAINT*; program(s) used to solve structure: *SHELXS97* (Sheldrick, 2008 ▶); program(s) used to refine structure: *SHELXL97* (Sheldrick, 2008 ▶); molecular graphics: *SHELXTL* (Sheldrick, 2008 ▶); software used to prepare material for publication: *SHELXTL*.

## Supplementary Material

Crystal structure: contains datablocks I, global. DOI: 10.1107/S1600536811016989/aa2005sup1.cif

Structure factors: contains datablocks I. DOI: 10.1107/S1600536811016989/aa2005Isup2.hkl

Additional supplementary materials:  crystallographic information; 3D view; checkCIF report

## Figures and Tables

**Table 1:** Hydrogen-bond geometry (Å, °)

*D*—H⋯*A*	*D*—H	H⋯*A*	*D*⋯*A*	*D*—H⋯*A*
C3—H3*A*⋯O1^i^	0.96	2.37	3.145 (2)	138
C2—H2*A*⋯Cl1^ii^	0.96	2.89	3.836 (3)	170

Symmetry codes: (i) 
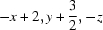
; (ii) 
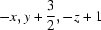
.

**Fig. 1.**
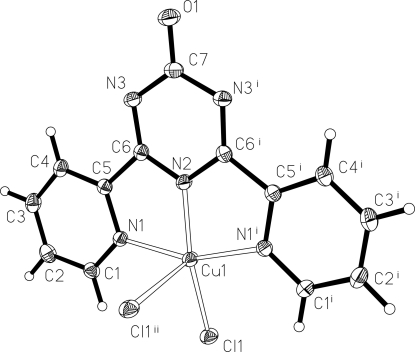
View of the title compound with the atom-labelling scheme. Displacement ellipsoids are drawn at the 30% probability level. [Symmetry codes: (i): x, 0.5 - y, z; (ii): 1 + x, y, z].

**Fig. 2.**
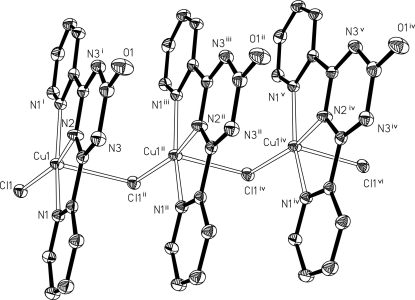
The chain structure in the title compound, H atoms are omitted for clarity. [Symmetry codes: (i): x, 0.5 - y, z; (ii): 1 + x, y, z; (iii): 1 + x, 0.5 - y, z; (iv): 2 + x, y, z; (v): 2 + x, 0.5 - y, z; (vi): 3 + x, y, z].

## supplementary crystallographic information

### Comment

The rigid multidentate polypyridyl ligands containing a triazine ring as a bridge have attracted greatly our attention due to their coordination diversity (Zhou, Li, Zheng *et al.*, 2006; Zhou, Li, Wu *et al.* 2006). Although coordination chemistry of the symmetrical ligands like TPT has been well explored, the observations on the asymmetric ligands containing triazine ring are still rare. As a contribution to the synthesis and structural studies of coordination abilities of 4,6-bis(2-pyridyl)-1,3,5-triazin-2-ol ligand (Wieprecht *et al.*, 2005; Cao *et al.*, 2008, 2009), I present here the crystal structure of the title compound (Fig. 1)- a new copper(II) complex with the ligand. Within the title compound, the copper(II) center is five-coordinated respectively by three N atoms [the distances of Cu—N are in the range of 1.896 (2) - 2.049 (2) Å] from the ligand and two Cl atoms [the bond lengths of Cu—Cl are 2.224 (1) Å and 2.778 (2) Å]. The complex is a chain structure, in which the ligand adopts a tridentate tripyridyl coordination mode and the chloride ion acts as a bridge (Fig. 2). The chains are linked *via* weak hydrogen bondings of C—H···O and C—H···Cl into a three-dimensional supramolecular network.

### Experimental

The ligand 4,6-bis(2-pyridyl)-1,3,5-triazin-2-ol was prepared according to previously reported procedures (Wieprecht *et al.*, 2005), yield 56%. The ligand (0.125 g, 0.5 mmol), CuCl_2_(0.07 g, 0.5 mmol), 7 ml distilled water and 7 ml ethanol were put into a 25 ml Teflon-lined Parr. The mixture was heated to 100 °C for 48 h, and then cooled to room temperature at a rate of 5 °C/h. The obtained mixture was filtered and green crystals were obtained. Yield: 0.108 g, 62% (base on the ligand). Anal. Calcd. for C_13_H_8_ClCuN_5_O (%): C 44.71, H 2.31, N 20.05; Found(%): C 44.52, H 2.43, N 19.91

### Refinement

All H atoms were calculated geometrically and treated as riding with C—H = 0.96 Å and *U*_iso_(H) = 1.2 *U*_eq_(C).

### Crystal data

**Table:**

[Cu(C_13_H_8_N_5_O)Cl]	*F*(000) = 350
*M**_r_* = 349.23	*D*_x_ = 1.898 Mg m^−^^3^
Monoclinic, *P*2_1_/*m*	Mo *K*α radiation, λ = 0.71073 Å
Hall symbol: -P 2yb	Cell parameters from 1109 reflections
*a* = 3.7687 (13) Å	θ = 2.3–25.0°
*b* = 13.698 (5) Å	µ = 2.01 mm^−^^1^
*c* = 11.852 (4) Å	*T* = 293 K
β = 92.851 (6)°	Block, green
*V* = 611.1 (4) Å^3^	0.09 × 0.09 × 0.07 mm
*Z* = 2	

### Data collection

**Table:**

Bruker SMART APEX CCD area-detector diffractometer	1109 independent reflections
Radiation source: fine-focus sealed tube	952 reflections with *I* > 2σ(*I*)
graphite	*R*_int_ = 0.030
φ and ω scans	θ_max_ = 25.0°, θ_min_ = 2.3°
Absorption correction: multi-scan (*SADABS*; Sheldrick, 1996)	*h* = −4→4
*T*_min_ = 0.840, *T*_max_ = 0.872	*k* = −16→16
3063 measured reflections	*l* = −14→13

### Refinement

**Table:**

Refinement on *F*^2^	Primary atom site location: structure-invariant direct methods
Least-squares matrix: full	Secondary atom site location: difference Fourier map
*R*[*F*^2^ > 2σ(*F*^2^)] = 0.033	Hydrogen site location: inferred from neighbouring sites
*wR*(*F*^2^) = 0.085	H-atom parameters constrained
*S* = 1.03	*w* = 1/[σ^2^(*F*_o_^2^) + (0.0528*P*)^2^] where *P* = (*F*_o_^2^ + 2*F*_c_^2^)/3
1109 reflections	(Δ/σ)_max_ < 0.001
103 parameters	Δρ_max_ = 0.49 e Å^−^^3^
0 restraints	Δρ_min_ = −0.27 e Å^−^^3^

### Special details

**Table:**

Geometry. All e.s.d.'s (except the e.s.d. in the dihedral angle between two l.s. planes) are estimated using the full covariance matrix. The cell e.s.d.'s are taken into account individually in the estimation of e.s.d.'s in distances, angles and torsion angles; correlations between e.s.d.'s in cell parameters are only used when they are defined by crystal symmetry. An approximate (isotropic) treatment of cell e.s.d.'s is used for estimating e.s.d.'s involving l.s. planes.
Refinement. Refinement of *F*^2^ against ALL reflections. The weighted *R*-factor *wR* and goodness of fit *S* are based on *F*^2^, conventional *R*-factors *R* are based on *F*, with *F* set to zero for negative *F*^2^. The threshold expression of *F*^2^ > σ(*F*^2^) is used only for calculating *R*-factors(gt) *etc*. and is not relevant to the choice of reflections for refinement. *R*-factors based on *F*^2^ are statistically about twice as large as those based on *F*, and *R*- factors based on ALL data will be even larger.

### Fractional atomic coordinates and isotropic or equivalent isotropic displacement parameters (Å^2^)

**Table:**

	*x*	*y*	*z*	*U*_iso_*/*U*_eq_	
Cu1	0.39787 (13)	0.2500	0.32504 (4)	0.0267 (2)	
Cl1	0.0169 (3)	0.2500	0.46244 (8)	0.0325 (3)	
O1	1.0547 (10)	0.2500	−0.1071 (3)	0.0457 (9)	
N1	0.4310 (6)	0.39688 (17)	0.29470 (19)	0.0252 (5)	
N2	0.6066 (9)	0.2500	0.1827 (3)	0.0288 (8)	
N3	0.8356 (7)	0.33967 (17)	0.03443 (19)	0.0295 (6)	
C1	0.3326 (8)	0.4686 (2)	0.3618 (3)	0.0294 (7)	
H1A	0.2282	0.4523	0.4316	0.035*	
C2	0.3786 (8)	0.5661 (2)	0.3345 (2)	0.0353 (8)	
H2A	0.3090	0.6168	0.3849	0.042*	
C3	0.5252 (9)	0.5888 (2)	0.2342 (3)	0.0357 (8)	
H3A	0.5538	0.6559	0.2130	0.043*	
C4	0.6294 (8)	0.5153 (2)	0.1635 (2)	0.0304 (7)	
H4A	0.7350	0.5298	0.0934	0.036*	
C5	0.5795 (7)	0.4205 (2)	0.1963 (2)	0.0249 (7)	
C6	0.6831 (8)	0.3335 (2)	0.1299 (2)	0.0249 (6)	
C7	0.9152 (11)	0.2500	−0.0159 (3)	0.0308 (10)	

### Atomic displacement parameters (Å^2^)

**Table:**

	*U*^11^	*U*^22^	*U*^33^	*U*^12^	*U*^13^	*U*^23^
Cu1	0.0324 (3)	0.0226 (3)	0.0256 (3)	0.000	0.0083 (2)	0.000
Cl1	0.0327 (6)	0.0348 (6)	0.0312 (6)	0.000	0.0117 (4)	0.000
O1	0.063 (2)	0.0376 (19)	0.0392 (19)	0.000	0.0247 (16)	0.000
N1	0.0264 (14)	0.0236 (13)	0.0257 (12)	−0.0008 (10)	0.0025 (10)	0.0006 (10)
N2	0.042 (2)	0.0183 (18)	0.0272 (19)	0.000	0.0114 (16)	0.000
N3	0.0319 (15)	0.0318 (15)	0.0254 (13)	−0.0008 (11)	0.0069 (11)	0.0019 (10)
C1	0.0306 (17)	0.0287 (17)	0.0290 (15)	0.0014 (13)	0.0031 (12)	−0.0039 (12)
C2	0.0368 (19)	0.0290 (17)	0.0400 (19)	0.0052 (14)	0.0000 (15)	−0.0083 (14)
C3	0.039 (2)	0.0246 (17)	0.0434 (19)	−0.0013 (13)	−0.0019 (15)	0.0045 (13)
C4	0.0325 (18)	0.0238 (16)	0.0347 (17)	−0.0035 (13)	0.0011 (14)	0.0032 (12)
C5	0.0224 (16)	0.0266 (16)	0.0255 (15)	0.0002 (12)	−0.0012 (12)	0.0005 (11)
C6	0.0222 (15)	0.0254 (16)	0.0268 (15)	−0.0009 (12)	−0.0020 (12)	0.0016 (12)
C7	0.030 (2)	0.038 (3)	0.024 (2)	0.000	0.0038 (18)	0.000

### Geometric parameters (Å, °)

**Table:**

Cu1—N2	1.896 (3)	N3—C7	1.405 (3)
Cu1—N1^i^	2.049 (2)	C1—C2	1.387 (4)
Cu1—N1	2.049 (2)	C1—H1A	0.9602
Cu1—Cl1	2.2239 (12)	C2—C3	1.371 (4)
Cu1—Cl1^ii^	2.778 (2)	C2—H2A	0.9602
O1—C7	1.225 (5)	C3—C4	1.380 (4)
N1—C1	1.329 (4)	C3—H3A	0.9603
N1—C5	1.358 (4)	C4—C5	1.371 (4)
N2—C6^i^	1.343 (3)	C4—H4A	0.9602
N2—C6	1.343 (3)	C5—C6	1.491 (4)
N3—C6	1.297 (4)	C7—N3^i^	1.405 (3)
			
N2—Cu1—N1^i^	79.18 (7)	C3—C2—C1	118.8 (3)
N2—Cu1—N1	79.18 (7)	C3—C2—H2A	120.6
N1^i^—Cu1—N1	158.25 (13)	C1—C2—H2A	120.6
N2—Cu1—Cl1	164.32 (11)	C2—C3—C4	120.0 (3)
N1^i^—Cu1—Cl1	100.12 (7)	C2—C3—H3A	120.0
N1—Cu1—Cl1	100.12 (7)	C4—C3—H3A	120.0
Cl1^ii^—Cu1—Cl1	100.12 (7)	C5—C4—C3	118.2 (3)
Cl1^ii^—Cu1—N1	100.12 (7)	C5—C4—H4A	120.6
Cl1^ii^—Cu1—N2	100.12 (7)	C3—C4—H4A	121.2
C1—N1—C5	118.5 (3)	N1—C5—C4	122.5 (3)
C1—N1—Cu1	126.9 (2)	N1—C5—C6	113.1 (2)
C5—N1—Cu1	114.60 (19)	C4—C5—C6	124.3 (3)
C6^i^—N2—C6	116.9 (3)	N3—C6—N2	125.2 (3)
C6^i^—N2—Cu1	121.53 (17)	N3—C6—C5	123.2 (2)
C6—N2—Cu1	121.53 (17)	N2—C6—C5	111.5 (3)
C6—N3—C7	115.3 (2)	O1—C7—N3	119.01 (17)
N1—C1—C2	122.1 (3)	O1—C7—N3^i^	119.01 (17)
N1—C1—H1A	118.8	N3—C7—N3^i^	122.0 (3)
C2—C1—H1A	119.1		

Symmetry codes: (i) *x*, −*y*+1/2, *z*; (ii) *x*+1, *y*, *z*.

### Hydrogen-bond geometry (Å, °)

**Table:**

*D*—H···*A*	*D*—H	H···*A*	*D*···*A*	*D*—H···*A*
C3—H3A···O1^iii^	0.96	2.37	3.145 (2)	138
C2—H2A···Cl1^iv^	0.96	2.89	3.836 (3)	170

Symmetry codes: (iii) −*x*+2, *y*+3/2, −*z*; (iv) −*x*, *y*+3/2, −*z*+1.
